# Prospective associations of dietary carbohydrate, fat, and protein intake with β-cell function in the CODAM study

**DOI:** 10.1007/s00394-018-1644-y

**Published:** 2018-03-10

**Authors:** Louise J. C. J. den Biggelaar, Simone J. P. M. Eussen, Simone J. S. Sep, Andrea Mari, Ele Ferrannini, Marleen M. van Greevenbroek, Carla J. van der Kallen, Casper G. Schalkwijk, Ilja C. W. Arts, Coen D. A. Stehouwer, Pieter C. Dagnelie

**Affiliations:** 1 Department of Epidemiology, Maastricht University, P.O. Box 616, 6200 MD Maastricht, The Netherlands; 2 School for Cardiovascular Diseases (CARIM), Maastricht University, Maastricht, The Netherlands; 3 Care and Public Health Research Institute (CAPHRI), Maastricht University, Maastricht, The Netherlands; 4 Centre of Expertise in Rehabilitation and Audiology, Adelante, Hoensbroek, The Netherlands; 5 CNR Institute of Clinical Physiology, Pisa, Italy; 6 CNR Institute of Neuroscience, Padova, Italy; 7 Department of Internal Medicine, Maastricht University Medical Centre, Maastricht, The Netherlands; 8 Maastricht Centre for Systems Biology (MaCSBio), Maastricht University, Maastricht, The Netherlands

**Keywords:** β-Cell function, Carbohydrate, Fat, Protein, Type 2 diabetes mellitus

## Abstract

**Purpose:**

Type 2 diabetes mellitus (T2DM) is characterized by both impaired pancreatic β-cell function (BCF) and insulin resistance. In the etiology of T2DM, BCF basically determines whether a person with a certain degree of insulin resistance develops T2DM, as β-cells are able to compensatorily increase insulin secretion. The effects of dietary intake on BCF are largely unknown. Our study aim was to investigate whether dietary macronutrient intake predicts BCF.

**Methods:**

Prospective data (median follow-up 7 years) of 303 individuals recruited from the CODAM study population (aged 40–70 years, 39% women) were analyzed. BCF was measured by C-peptide deconvolution and physiological modeling of data from a 5-point, 75-g, 2-h oral glucose tolerance test. Macronutrient intake was estimated by a 178-item Food Frequency Questionnaire.

**Results:**

Associations adjusted for relevant covariates of baseline macronutrient intake with model-derived parameters describing BCF (glucose sensitivity, rate sensitivity or potentiation) or C-peptidogenic index were detected for *trans* fat [standardized regression coefficient (95%-CI) glucose sensitivity − 0.14 (− 0.26, − 0.01)] per g, cholesterol [potentiation 0.20 (0.02, 0.37)] per 100 mg, dietary fiber [glucose sensitivity 0.21 (0.08, 0.33)] per 10 g, MUFA glucose sensitivity 0.16 (0.02, 0.31) per 10 g, and polysaccharide [potentiation − 0.24 (− 0.43, − 0.05), C-peptidogenic index − 0.16 (− 0.29 − 0.03); odds ratio lowest versus highest tertile (95%-CI) rate sensitivity 1.51 (1.06, 2.15)) per 50 g.

**Conclusions:**

In this population at high risk for developing T2DM, polysaccharide and *trans* fat intake were associated with worse BCF, whereas increased intake of MUFA, dietary cholesterol, and fiber were associated with better BCF.

**Electronic supplementary material:**

The online version of this article (10.1007/s00394-018-1644-y) contains supplementary material, which is available to authorized users.

## Introduction

The number of people with type 2 diabetes mellitus (T2DM) is expected to rise from 366 million worldwide in 2011 to 552 million in 2030 [1]. This enormous rise in T2DM prevalence will most likely be accompanied by an increase in T2DM complications and associated diseases, including cardiovascular disease, neuropathy, nephropathy, and retinopathy, and reduced life expectancy [2–4]. Lifestyle modification, including eating healthier diets, has been suggested to reduce the risk of developing T2DM by 40–70% [5]. A reduced intake of total fat, saturated fat, *trans* fat, cholesterol, mono- and disaccharides, and an increased intake of fiber, MUFA and PUFA is recommended by several organizations, including the American Diabetes Association [6], Canadian Diabetes Association [7] and Diabetes and Nutrition Study Group of the European Association for the Study of Diabetes [8].

Results of a meta-analysis [9] of prospective cohort studies showed associations that are not in line with current dietary recommendations regarding total fat, saturated fat, MUFA, n-6 PUFA, total protein and vegetable protein. Furthermore, results were inconsistent for total carbohydrate (positive versus no association), *trans* fat (positive versus negative versus no association), n-3 PUFA (negative versus no association) and animal protein (positive versus no association) [9]. Moreover, evidence is inconsistent regarding the association of polysaccharide, mono- and disaccharide, and dietary fiber intake with T2DM (not evaluated in the meta-analysis) [10–13]. Although, observational studies are not an ideal approach to show causality, they can provide a good indication of the associations between dietary intake and T2DM.

These inconsistencies in results between studies may be caused by among others heterogeneity in study populations, study design, dietary assessment, and statistical analyses [14, 15]. Furthermore, T2DM represents the final stage of glucose metabolism impairmen. Besides as T2DM is a dichotomous outcome measure, it reduces the power of statistical analyses. Reduced β-cell function (BCF) can be considered as the critical determinant for the development of prediabetes and T2DM because β-cells can compensate for a decreased insulin sensitivity by upregulating insulin secretion and thereby prevent the development of T2DM. However, in the condition of reduced insulin sensitivity and decreased BCF, in which β-cells are not able to secrete sufficient insulin, hyperglycemia arises, which eventually results in prediabetes or T2DM [16, 17].

The use of BCF, as an outcome measure in epidemiological studies, has several methodological advantages. First of all, it provides physiological information on early abnormalities in glucose metabolism which eventually result in prediabetes and T2DM development. Furthermore, as BCF is a continuous measure, the issue of misclassifications of subjects with borderline blood glucose value is not relevant. Moreover, significant changes in BCF not resulting in the development of T2DM are taken into account, which is important as the impairment of BCF starts already in the normal glucose metabolism range [17]. The last two mentioned advantages increase the statistical power to detect associations [18].

So far, few studies evaluated the association between macronutrient intake and BCF in adults. These were cross-sectional studies [19–23] which focused on a limited selection of macronutrients, including total carbohydrate, total fat, saturated fat, MUFA, total PUFA, and dietary fiber, mainly in relation to early-phase insulin secretion (i.e. starting almost immediately after ingestion of a meal and inhibits glucose release by the liver). Based on the recommendations of diabetes organizations [6–8], we hypothesized an inverse association of saturated fat, *trans* fat, cholesterol, mono- and disaccharides intake with BCF, and a positive association of fiber, MUFA and PUFA intake with BCF. Therefore, the aim of this study was to investigate whether intake of dietary carbohydrate, fat and protein is prospectively associated with multiple components of BCF, e.g. early-phase insulin secretion, overall insulin secretion, β-cell glucose sensitivity, β-cell rate sensitivity, and β-cell potentiation factor, taking the degree of insulin sensitivity into account.

## Methods

### Study population and design

The current study used prospective data from the Cohort on Diabetes and Atherosclerosis Maastricht (CODAM) study. The CODAM study population consists of participants at high risk for the development of T2DM. Inclusion criteria were: age 40–70 years and either a BMI  ≥ 25 kg/m^2^, a family history for T2DM, a history of gestational diabetes, the use of antihypertensive medication, a postprandial blood glucose ≥ 6.0 mmol/L or glycosuria. The CODAM study has been previously described in detail [24]. Briefly, 574 individuals were extensively characterized at baseline [1999–2002] with regard to their lifestyle, and cardiovascular and metabolic profile during two visits to the University’s metabolic research unit. At the follow-up examination [2006–2009], with a median follow-up period of 7.0 years (IQR 6.9–7.1), the measurements were repeated in 491 individuals (overall attrition rate 14%). The CODAM study was approved by the Medical Ethics Committee of the Maastricht University Medical Center, and all subjects gave written informed consent. For the present analyses, exclusion criteria were no participation in follow-up measurements (*n* = 79), previously diagnosed diabetes (defined as self-reported diagnosis and/or use of glucose lowering medication) (*n* = 66), suffering from any type of cancer (*n* = 46), incomplete oral glucose tolerance test (OGTT) data (*n* = 45), unreliable FFQ data (defined as > 10% missing items in the FFQ) (*n* = 30) and implausible total energy intake (< 800 or > 4200 kcal/day for men and < 500 or > 3500 kcal/day for women) [25] (*n* = 5). This resulted in a final study population of 303 individuals.

### OGTT-derived measurements

Participants underwent an OGTT at baseline and after 7 years of follow-up. After an overnight fast (> 12 h), venous blood samples were collected before, and 15, 30, 60, and 120 min after ingestion of a 75 g oral glucose load. Plasma for the assessment of insulin and C-peptide was collected in EDTA tubes on ice, separated after centrifugation (3000×*g* for 15 min at 4 °C), and stored at − 80 °C until the assays were performed. The time between collection and storage was < 2 h. Insulin and C-peptide were measured by use of a custom duplex array of MesoScale Discovery (MesoScale Discovery, Gaithersburg, MD, USA, http://www.mesoscale.com). In short, 96 well-plates, with capture antibodies against insulin and C-peptide patterned on distinct spots in the same well, were supplied by the manufacturer. Samples (10 µL/well), detection antibodies and read buffer for electrochemiluminescence were applied according to manufacturer’s instruction and plates were read using a SECTOR® 2400 Imager. Detection ranges of the assay were 35−25,000 pg/mL for insulin and 70−50,000 pg/mL for C-peptide. Interassay coefficients of variation for insulin and C-peptide were 9.7 and 7.9%, respectively. Insulin and C-peptide values were converted from pg/mL to pmol/L using a molar mass of 5808 g for insulin and 3010 g for C-peptide. Plasma for the assessment of glucose was collected in NaF/KOx tubes on ice. Glucose was measured by use of the hexokinase method (HK-G6PD method; ABX Diagnostics Glucose HK 125, Montpellier, France).

Glucose metabolism status was defined according to the WHO 2006 criteria [26]: normal glucose metabolism (NGM; fasting plasma glucose < 6.1 mmol/L and 2 h post plasma glucose < 7.8 mmol/L), prediabetes (fasting plasma glucose levels between 6.1 and 7.0 mmol/L and/or 2 h post glucose levels between 7.8 and 11.1 mmol/L) and T2DM (fasting plasma glucose ≥ 7.0 mmol/L and/or 2 h post glucose ≥ 11.1 mmol/L).

### β-cell function

As BCF consists of multiple components, it cannot be described by a single measure. Therefore, we used three mathematical model-based parameters (β-cell glucose sensitivity, β-cell potentiation factor ratio and β-cell rate sensitivity) according to a previously described model [27], and two classic, relatively simple BCF-indices (C-peptidogenic index and the ratio of the C-peptide to glucose area under the curve). Only dynamic measures of BCF were included in the analyses, because these provide a more accurate reflection of BCF than basal measures. Furthermore, these dynamic measures reflect both basal and post-load insulin secretory responses [28, 29].

The first mathematical model parameter ‘β-cell glucose sensitivity’ is the slope of the glucose-insulin secretion dose–response function [27], and represents the dependence of insulin secretion on absolute glucose concentration at any time point during the OGTT. β-cell glucose sensitivity is a sensitive index to quantify β-cell dysfunction [17, 30, 31]. The dose–response relationship is modulated by β-cell potentiation, which accounts for higher insulin secretion during the descending phase of hyperglycaemia than during the ascending phase of an OGTT, for the same glucose concentration. The second parameter β-cell potentiation is set as a positive function of time and averages 1 during the OGTT. Therefore, it represents the relative potentiation of the insulin secretion response to glucose. The β-cell potentiation parameter used in the present analysis represents the ratio of the β-cell potentiation factor at the end of the 2-h OGTT relative to the β-cell potentiation factor at the start. The third parameter ‘β-cell rate sensitivity’ is a marker of early phase insulin release, and represents the dynamic dependence of insulin secretion on the rate of change in glucose concentration [27].

The simple BCF-indices C-peptidogenic index (ΔCP_30_/Δ*G*_30_) and the ratio of the C-peptide to glucose area under the curve (CP_AUC_/*G*_AUC_) were also calculated. The C-peptidogenic index (the equivalent of the insulinogenic index) reflects early phase insulin secretion and has good discriminatory ability to predict (pre)T2DM (ROC AUCs ≥ 78%) [32].

### Dietary intake

Habitual dietary intake over the past 12 months was estimated by a semi-quantitative 178 food item food frequency questionnaire (FFQ) [33, 34]. Briefly, for each food item, frequency (ranging from ‘never or less than once a month’ to ‘every day’) and amount was noted by the participants in a closed answer format. Total energy and macronutrient intake were computed using the extended version of the Dutch food composition table (NEVO) of 2001 [35].

The food items in the FFQ were selected using the Dutch National Food Consumption Survey 1987–1988 dataset. Products that accounted for at least 90% of the population mean intake of macronutrients and food groups were selected [33]. According to the Dutch National Food Consumption Survey (1997–1998), the main product groups of macronutrient intake in the Dutch population were as follows: For protein intake, the main product groups were meat and poultry, followed by milk products. For saturated fatty acids, dairy (including milk products and cheese), followed by fats, oils, and savoury sauces, and for unsaturated fatty acids the product group fats, oil and savoury sauces, followed by nuts, seeds, and snacks, meat and poultry. For mono- and disaccharides the main sources were sugar, sweets, and sweet sauces, followed by milk products, sugar-sweetened beverages and fruit, and for polysaccharides, bread, followed by potatoes and cereal products.

### Anthropometric and other measurements

Body height (cm) and weight (kg) were measured to the nearest 1 cm and 0.1 kg with the participants wearing light clothing and no shoes [24]. Waist circumference, blood pressure, the presence of a history of cardiovascular disease (CVD) and blood lipid profiles, including triglycerides, total cholesterol, HDL and LDL cholesterol, were determined as described previously [36, 37]. Smoking status (never, ex, or current smoker), presence of cancer and medication use were self-reported [38]. Finally, physical activity level (min/week × intensity) was measured by the validated Short Questionnaire to Assess Health-enhancing Physical Activity (SQUASH) [39].

### Statistical analysis

All analyses were performed using the software package SPSS Statistics version 23.0 for Windows (SPSS, IBM Corp, Armonk, NY, USA).

Study population characteristics were described by medians (25th-75th percentiles) or number of participants per category (% of study population). β-cell potentiation factor and the C-peptidogenic index were log-transformed as these were not normally distributed.

Linear regression analyses were performed to assess associations between macronutrient intake at baseline and BCF at follow-up. Associations were presented as standardized betas (βs) per 100 mg, 1 g, 10 g or 50 g increment for macronutrient intake, depending on the amount of the specific macronutrient intake in the CODAM population. As the distribution of β-cell rate sensitivity could not be normalized by transformations, β-cell rate sensitivity was categorized into tertiles. Multinomial regression analyses were performed for β-cell rate sensitivity, with the highest tertile [i.e. participants with the best β-cell rate sensitivity) as the reference category, and presented as odds ratios (OR (95% CI)].

Macronutrients were energy-adjusted using the residual method of Willett et al. [40] and additionally adjusted for total energy intake. Furthermore, continuous BCF measures at follow-up were adjusted for BCF at baseline (derived from a 2 h, 4-points OGTT including 0, 30, 60, and 120 min), by including baseline BCF as covariate in the regression models. In addition, to assess BCF relative to the prevailing level of insulin resistance, the Matsuda index (10,000/√*G*_0_ × *I*_0_ × mean *G* × mean *I*) [41], was included as a covariate in the regression models.

Potential confounders were included as covariates in the regression models if the regression coefficient of at least one BCF-index changed by > 10%. Accordingly, model 1 was adjusted for insulin resistance, age, and sex. Model 2 was further adjusted for BMI, mean arterial blood pressure, anti-hypertensive medication, lipid-modifying medication, family history of T2DM, total energy intake, and intake of dietary fiber, polysaccharide (except for the regression analysis of total carbohydrate), and MUFA (except for the regression analysis of total fat). Based on our data, total physical activity, CVD, smoking status, and the intake of alcohol, saturated fat, *trans* fat, n-3 and n-6 PUFA, mono- and disaccharides, animal protein and vegetable protein had no confounding effects on the associations of macronutrient intake with BCF, and were, therefore, not included as confounders.

## Results

### Population characteristics

The demographics of the total CODAM population (*n* = 574) and of the individuals included in the analyses of the current study (*n* = 303) are shown in Table 1. The median age of the final study population was 58.8 years, and 61.7% were men. Altogether, almost 80% of the participants were overweight (56.8%) or obese (22.2%). NGM, prediabetes, and newly diagnosed T2DM were present in 65.3%, 26.1%, and 8.6% of the final study population, respectively. During the follow-up period, 53 NGM persons (26.8%) developed prediabetes and 15 NGM persons (7.6%) developed T2DM. Furthermore, 32 prediabetic persons (40.5%) developed T2DM.

**Table 1:** Demographics

	Total CODAM population (*n* = 574)				Analysis population (*n* = 303)			
	Median	*N*	95% CI	%	Median	*N*	95%CI	%
Demography								
Age	60.3		46.7–70.4		58.8		45.2–68.5	
Sex [male]		352		61.3		187		61.7
Health								
BMI								
< 25 kg/m^2^		104		18.1		63		20.8
25–30 kg/m^2^		292		50.9		172		56.8
> 30 kg/m^2^		177		30.8		67		22.2
Glucose metabolism status								
NGM [*n*]		301		52.8		198		65.3
prediabetes [*n*]		127		22.3		79		26.1
Newly diagnosed T2DM [*n*]		80		14.0		26		8.6
Previously diagnosed T2DM [*n*]		62		10.9		n/a		n/a
CVD (yes)		158		27.6		44		14.5
Lipid-modifying medication (yes)		108		18.8		48		15.8
Systolic blood pressure (mmHg)	138		112–176		134		111–166	
Diastolic blood pressure (mmHg)	81.5		67.5–98.0		80.5		67.0–97.7	
Anti-hypertensive medication (yes)		220		38.3		91		30.0
Lifestyle								
Smoking								
Never		164		28.6		95		31.4
Ex		283		49.3		143		47.2
Current		114		19.9		60		19.8
Unknown		13		2.3		3		0.7
Total PA (min/week × intensity) × 10^3^	5.85		1.34–15.1		6.07		1.48–14.9	
Blood parameters								
Fasting glucose (mmol/L)	5.60		4.76–9.22		5.44		4.74–7.08	
2-h glucose (mmol/L)	6.73		3.75–15.7		6.33		3.60–11.8	
Fasting insulin (pmol/L)	74.0		30.0–247		63.2		28.5–187	
2-h insulin (pmol/L)	460		112–1855		441		106–1391	
HBA1C (%)	5.80		5.10–7.46		5.70		5.00–6.60	
Total cholesterol (mmol/L)	5.20		3.68–6.80		5.20		3.72–6.80	
HDL cholesterol (mmol/L)	1.14		0.72–1.85		1.18		0.77–1.93	
LDL cholesterol (mmol/L)	3.30		1.90–4.80		3.40		1.90–4.89	
Triglycerides (mmol/L)	1.40		0.70–3.30		1.40		0.60–3.28	
BCF-indices								
β-cell glucose sensitivity (pmol/min/m^2^/mM)	35.1		9.67–86.6		37.5		13.1–74.3	
β-cell potentiation factor	1.12		0.89–3.71		1.13		0.92–3.78	
Β-cell rate sensitivity pmol/m^2^/mM	78.1		0.00–928		85.7		0.00–972	
∆CP_30_/∆G_30_	361		109–373		384		130–9258	
CP_AUC_/G_AUC_	218		109–906		218		117–376	
Insulin sensitivity								
Matsuda index	2.95		0.85–6.92		3.24		0.96–7.27	

*n* number, *BMI* Body Maas Index, *NGM* normal glucose metabolism, *T2DM* newly diagnosed type 2 diabetes mellitus, *n/a* not applicable, *CVE* self-reported cardiovascular event, *PA* physical activity, *t*_*x*_ measured x minutes after the start of the oral glucose tolerance test, *HbA1C* glycated haemoglobin, *HDL* High-density lipoprotein, *LDL* low-density lipoprotein, *DI* disposition index

Relative to the total study population, the analysis population included smaller proportions of individuals with obesity, prediabetes, T2DM, CVD, lipid-lowering and blood pressure lowering medication, and overall individuals who were more physically active and had a slightly better mean BCF (Table 1).

The consumption of carbohydrate, fat, protein, and alcohol, in the analysis population, was on average 44.8, 34.9, 15.6, and 4.7 En%, respectively. Macronutrient intake was essentially the same for the analysis population and the total study population (Table 2).

**Table 2:** Mean macronutrient intake at baseline

	Total CODAM population (*n* = 574)				Analysis population (*n* = 303)		
	Mean		SD	EN(%)	Mean	SD	EN(%)
Total energy intake (kcal)	2205	669			2243	678	
Total carbohydrate (g/day)	241	76.4		44.5	244	77.0	44.4
Polysaccharide (g/day)	135	45.8			138	46.2	
Mono- and disaccharide (g)	105	42.2			106	42.2	
Fiber (g)	25.2	7.36			25.3	7.11	
Total protein (g)	84.3	23.3		15.8	85.0	23.3	15.6
Animal protein (g)	51.3	16.9			51.3	16.9	
Vegetable protein (g)	33.0	10.3			33.8	10.6	
Total fat (g)	89.2	33.7		35.3	90.9	34.6	35.3
Saturated fat (g/d)	34.1	13.4			34.8	13.9	
*trans* fat (g/d)	3.20	1.58			3.26	1.50	
MUFA (g/d)	28.7	11.6			29.2	11.9	
N-3 PUFA (g/day)	1.48	0.65			1.51	0.64	
N-6 PUFA (g/day)	13.9	6.14			14.2	6.32	
Cholesterol (mg/day)	242	94.9			243	93.4	
Alcohol (g/day)	14.7	17.7		5.30	15.4	17.5	4.60

*g* gram, *EN%* energy percent

### Associations between baseline macronutrient intake and BCF after 7 years of follow-up

Polysaccharide intake was associated with worse β-cell potentiation factor, C-peptidogenic index [βs − 0.24 (− 0.43, − 0.05) and − 0.16 (− 0.29,− 0.03), respectively, per 50 g in fully adjusted models] and with worse β-cell rate sensitivity [OR 1.51 (95% CI 1.06, 2.15) per 50 g for the first versus the third tertile (i.e. reference group which has the best β-cell rate sensitivity)]. Furthermore, polysaccharide intake was associated with worse β-cell glucose sensitivity and overall insulin secretion, but non-significantly. Mono- and disaccharide intake were associated with better β-cell rate sensitivity in model 1 only [OR 0.76 (95% CI 0.60, 0.96) per 50 g for the first versus the third tertile (i.e. reference group which has the best β-cell rate sensitivity)] (Tables 3, 4).

**Table 3:** Associations between macronutrient intake and mathematical model BCF parameters

	Unit		β-cell glucose sensitivity		β-cell potentiation factor		β-cell rate sensitivity^a^			
							Tertiles			
			*n* = 303		*n* = 303		First (*n* = 101) ≤ 148.40 pmol/m^2^/mM		Second (*n* = 101) 148.40–348.77 pmol/m^2^/mM	
			β^b^	95% CI	β^b^	95% CI	OR^c^	95% CI	OR^c^	95% CI
Total carbohydrate	50 g	Model 1	− 0.07	− 0.17, 0.04	− 0.08	− 0.21, 0.04	0.94	0.81, 1.11	1.03	0.89,1.19
		Model 2	− 0.03	− 0.22, 0.17	− 0.09	− 0.34, 0.15	0.98	0.72, 1.35	1.26	0.92, 1.71
Polysaccharide	50 g	Model 1	− 0.05	− 0.16, 0.06	− 0.18*	− 0.31, − 0.05	1.18	0.93, 1.50	1.02	0.80, 1.28
		Model 2	− 0.08	− 0.22, 0.07	− 0.24*	− 0.43, − 0.05	1.51*	1.06, 2.15	1.03	0.74, 1.44
Mono- and disaccharide	50 g	Model 1	− 0.05	− 0.15, 0.06	0.03	− 0.09, 0.15	0.76*	0.60, 0.96	1.04	0.84, 1.28
		Model 2	0.01	− 0.14, 0.16	0.01	− 0.17, 0.19	0.82	0.57, 1.17	1.26	0.91, 1.75
Fiber	10 g	Model 1	0.08	− 0.03, 0.18	− 0.09	− 0.21, 0.04	0.97	0.75, 1.27	1.04	0.80, 1.35
		Model 2	0.21*	0.08, 0.33	0.01	− 0.16, 0.17	0.85	0.59, 1.21	1.14	0.81, 1.60
Total protein	10 g	Model 1	− 0.01	− 0.12, 0.09	0.01	− 0.11, 0.14	0.97	0.87, 1.07	0.99	0.89, 1.10
		Model 2	− 0.04	− 0.18, 0.10	0.02	− 0.15, 0.20	0.99	0.85, 1.15	1.03	0.89, 1.18
Animal protein	10 g	Model 1	− 0.03	− 0.13, 0.08	0.10	− 0.03, 0.23	0.89	0.66, 1.20	0.99	0.74, 1.31
		Model 2	− 0.05	− 0.18, 0.07	0.06	− 0.10, 0.22	0.96	0.85, 1.07	1.00	0.90, 1.11
Vegetable protein	10 g	Model 1	0.02	− 0.09, 0.13	− 0.18*	− 0.30, − 0.05	0.99	0.86, 1.14	1.03	0.90, 1.18
		Model 2	0.12	− 0.10, 0.33	− 0.24	− 0.50, 0.03	0.97	0.59, 1.61	0.90	0.55, 1.45
Total fat	50 g	Model 1	0.00	− 0.11, 0.11	− 0.01	− 0.14, 0.11	0.86	0.57, 1.30	0.98	0.66, 1.44
		Model 2	0.10	− 0.07, 0.26	− 0.06	− 0.26, 0.15	1.28	0.65, 2.53	1.43	0.76, 2.71
Saturated fat	10 g	Model 1	− 0.05	− 0.16, 0.05	0.01	− 0.12, 0.13	0.95	0.79, 1.15	1.08	0.91, 1.28
		Model 2	− 0.16	− 0.33, 0.01	− 0.01	− 0.23, 0.21	1.09	0.77, 1.55	1.52*	1.08, 2.12
*trans* fat	1 g	Model 1	− 0.12*	− 0.23, − 0.01	− 0.02	− 0.14, 0.11	0.94	0.81, 1.09	1.03	0.91, 1.18
		Model 2	− 0.14*	− 0.26, − 0.01	0.08	− 0.08, 0.25	0.88	0.73, 1.07	1.11	0.93, 1.31
MUFA	10 g	Model 1	0.05	− 0.06, 0.16	0.00	− 0.12, 0.13	0.90	0.72, 1.14	0.93	0.75, 1.17
		Model 2	0.16*	0.02, 0.31	− 0.05	− 0.22, 0.13	1.12	0.79, 1.58	1.07	0.77, 1.48
N-3 PUFA	1 g	Model 1	0.07	− 0.03, 0.17	− 0.04	− 0.16, 0.07	0.17	0.01, 3.24	0.02*	0.00, 0.38
		Model 2	0.04	− 0.07, 0.15	− 0.04	− 0.17, 0.10	0.36	0.01, 11.45	0.01*	0.00, 0.44
N-6 PUFA	1 g	Model 1	0.07	− 0.04, 0.17	− 0.07	− 0.19, 0.05	0.99	0.96, 1.03	0.98	0.95, 1.02
		Model 2	0.04	− 0.08, 0.17	− 0.07	− 0.22, 0.09	1.01	0.96, 1.06	0.98	0.93, 1.02
Cholesterol	100 mg	Model 1	− 0.03	− 0.13, 0.08	0.13	− 0.01, 0.26	0.94	0.76, 1.16	0.91	0.74, 1.13
		Model 2	− 0.04	− 0.17, 0.09	0.20*	0.02, 0.37	0.91	0.68, 1.20	0.94	0.71, 1.25

Model 1: adjusted for age and sex, baseline BCF and follow-up insulin resistance

Model 2: adjusted for model 1 and BMI, mean arterial blood pressure, anti-hypertensive medication, lipid-modifying medication, family history of T2DM, total energy intake, and intake of dietary fiber, polysaccharide and MUFA

^a^Tertiles of rate sensitivity were not adjusted for baseline rate sensitivity

^b^Positive values mean an improvement of the specific BCF parameter

^c^Group 3 (the group with the best rate sensitivity > 349 pmol/m^2^/mM) is the reference group

*p value < 0.05

**Table 4:** Associations between macronutrient intake and simple BCF-indices

	Unit		C-peptidogenic index *n* = 292		CP_AUC_/*G*_AUC_*n* = 302	
			β^a^	95% CI	β^a^	95% CI
Total carbohydrate	50 g	Model 1	− 0.08	− 0.17,0.02	− 0.07	− 0.14,0.01
		Model 2	− 0.13	− 0.32,0.05	− 0.07	− 0.22,0.07
Polysaccharide	50 g	Model 1	− 0.12*	− 0.22,− 0.03	− 0.04	− 0.13,0.02
		Model 2	− 0.16*	− 0.29,− 0.03	− 0.04	− 0.15,0.06
Mono- and disaccharide	50 g	Model 1	0.00	− 0.09,0.09	− 0.04	− 0.15,0.06
		Model 2	− 0.04	− 0.18,0.10	− 0.04	− 0.14,0.06
Fiber	10 g	Model 1	− 0.03	− 0.12,0.07	− 0.02	− 0.09,0.05
		Model 2	0.08	− 0.05,0.20	0.04	− 0.06,0.13
Total protein	10 g	Model 1	− 0.01	− 0.11,0.09	0.00	− 0.07,0.08
		Model 2	0.01	− 0.12,0.14	0.00	− 0.07,0.08
Animal protein	10 g	Model 1	0.02	− 0.08,0.11	0.01	− 0.06,0.08
		Model 2	0.00	− 0.11,0.12	0.00	− 0.08,0.08
Vegetable protein	10 g	Model 1	− 0.06	− 0.16,0.04	− 0.10	− 0.08,0.06
		Model 2	0.03	− 0.18,0.23	0.01	− 0.10,0.13
Total fat	50 g	Model 1	− 0.01	− 0.10,0.09	0.00	− 0.07,0.08
		Model 2	− 0.01	− 0.11,0.09	0.04	− 0.08,0.15
Saturated fat	10 g	Model 1	− 0.03	− 0.12,0.07	− 0.02	− 0.09,0.06
		Model 2	− 0.08	− 0.24,0.09	− 0.07	− 0.19,0.06
*trans* fat	1 g	Model 1	0.00	− 010,0.09	− 0.04	− 0.13,0.05
		Model 2	0.05	− 0.07,0.16	− 0.04	− 0.13,0.05
MUFA	10 g	Model 1	0.01	− 0.09,0.11	0.02	− 0.06,0.09
		Model 2	0.00	− 0.14,0.14	0.06	− 0.05,0.16
N-3 PUFA	1 g	Model 1	0.03	− 0.06,0.12	0.06	− 0.02,0.14
		Model 2	0.03	− 0.08,0.13	0.06	− 0.02,0.14
N-6 PUFA	1 g	Model 1	0.03	− 0.06,0.12	0.03	− 0.04,0.11
		Model 2	0.04	− 0.08,0.16	0.04	− 0.05,0.13
Cholesterol	100 mg	Model 1	− 0.03	− 0.12,0.07	− 0.02	− 0.10,0.05
		Model 2	− 0.03	− 0.15,0.10	− 0.01	− 0.10,0.09

Model 1: adjusted for age and sex, baseline BCF and follow-up insulin resistance

Model 2: adjusted for model 1 and BMI, mean arterial blood pressure, anti-hypertensive medication, lipid-modifying medication, family history of T2DM, total energy intake, and intake of dietary fiber, polysaccharide and MUFA

^a^Positive values mean an improvement of the specific BCF parameter

Regarding fats, *trans* fat intake was associated with worse β-cell glucose sensitivity [βs of − 0.12 (− 0.23, − 0.01) and − 0.14 (− 0.26, − 0.01) per gram in age- and sex-adjusted, and fully adjusted models, respectively]. Furthermore, saturated fat was associated with worse β-cell rate sensitivity revealed [OR 1.52 (1.08–2.12) per 10 g for the second tertile compared with the third tertile (reference group)]. Besides, n-3 PUFA was associated with better β-cell rate sensitivity [OR of 0.01 (0.00, 0.44) per gram for the second tertile compared with the third tertile (reference group) in the fully adjusted model], and MUFA intake was associated with better β-cell glucose sensitivity [β 0.16 (0.02, 0.31) per 10 g in the fully adjusted model]. Furthermore, dietary cholesterol was associated with better β-cell potentiation factor [β 0.20 (0.02, 0.37) per 100 mg in the fully adjusted model] (Table 3).

Protein intake was not associated with any of the BCF parameters (Tables 3, 4).

Dietary fiber intake was associated with better β-cell glucose sensitivity [β 0.21 (0.08, 0.33) per 10 g in the fully adjusted model] (Table 3).

## Discussion

This is the first study focusing on the prospective association of carbohydrate, fat and protein intake with multiple measures of BCF. Results revealed that *trans* fat and polysaccharide intake were associated with worse BCF, and MUFA, dietary cholesterol and fiber intake with better BCF.

In our study, statistically significant associations were found for polysaccharide intake with worse of β-cell rate sensitivity, β-cell potentiation factor, and C-peptidogenic index. No associations of mono- and disaccharides intake with BCF could be detected in the fully adjusted models. Our findings suggest that a higher intake of polysaccharides may result in reduced early-phase insulin release, and impaired potentiation of insulin secretion to glucose, which may explain the associations of polysaccharide intake with increased risk of T2DM observed in some [12, 42, 43], but not all [10, 44], prospective studies. It is conceivable that these findings are caused by the quality of dietary carbohydrates. According to the Dutch National Food Consumption Survey (1997–1998), the main sources of polysaccharides in the Dutch population were potatoes, bread, and cereals. Bread and cereals often consist of refined carbohydrates. These refined carbohydrates and potatoes might influence glucose homeostasis via their high glycemic load [43, 45, 46]. A high glycaemic load causes high peaks in blood glucose and insulin concentrations thereby increasing T2DM risk [47]. Regarding mono- and disaccharide intake, a prospective study revealed a positive association of fructose and glucose intake with T2DM risk and a negative association of sucrose intake with T2DM risk [10]. Whether these mono- and disaccharides subcategories also have opposite associations with BCF, which in turn might explain the null association of mono- and disaccharide intake with BCF in the current study, should be further explored.

Regarding dietary fat intake, an inverse association was observed for *trans* fat with β-cell glucose sensitivity. Furthermore, positive associations were observed for MUFA intake with β-cell glucose sensitivity, and for dietary cholesterol intake with β-cell potentiation factor. On top of that, n-3 PUFA was associated with a lower risk of moderate impairment of β-cell rate sensitivity, as represented by the second tertile of rate sensitivity.

In a meta-analysis, only one study showed an association between *trans* fat intake and increased T2DM risk [9], which may be explained by the inverse association between *trans* fat intake and β-cell glucose sensitivity, as observed in the present study. The mechanisms underlying the negative association between *trans* fat intake and β-cell glucose sensitivity observed in our study are unclear, but it is suggested that *trans* fat intake is positively associated with several proinflammatory markers, which in turn may cause β-cell damage [48]. Of note, over the last decades *trans* fat intake declined strongly [49], therefore, the relevance of this finding for today is uncertain.

The absence of an association between saturated fat intake and BCF found the current study is in line with previous studies focused on BCF [19, 21] and a meta-analysis focused on T2DM [9]. However, a prospective study showed an association of even-chain saturated fatty acids with increased T2DM risk and an association of odd-chain saturated fatty acids with decreased T2DM risk [50]. Whether these saturated fat subcategories have opposite associations with BCF, like with T2DM (55), should be further explored. Potentially involved mechanisms are inflammation, glucolipotoxicity, endoplasmic reticulum stress and oxidative stress [19, 48, 51].

The positive association of MUFA intake with β cell glucose sensitivity found in our study is in line with the positive association of MUFA intake with fasting insulin secretion observed in a previous cross-sectional study [22]. It has been hypothesized that MUFA intake preserves or even enhances β-cell proliferation and acts anti-apoptotic [51], thereby improving BCF. This contrasts with the null association observed between MUFA intake and T2DM in a meta-analysis [9].

Evidence of the associations of n-3 and n-6 PUFA intake with T2DM is conflicting [9, 52–54]. A positive association of n-3 PUFA with BCF was found in animal and in vitro studies [53, 55]. These studies suggest that n-3 PUFA decreases the circulating levels of inflammatory cytokines and endoplasmic reticulum stress, which in turn will result in lower levels of β-cell apoptosis [53, 55]. Two randomized controlled trials showed no association of n-3 PUFA with early-phase BCF [56, 57]. One randomized controlled trial showed an inverse association of n-6 PUFA with the early-phase BCF disposition index [56]. The inverse association found in that trial is in line with the inverse association found between n-6 PUFA intake and the β-cell potentiation factor disposition index in our study. This might implicate that n-6 PUFA intake is primarily associated with worse insulin sensitivity. However, more studies investigating the association of n-3 and n-6 with BCF are required.

Finally, literature demonstrated an association between dietary cholesterol intake and increased risk of T2DM [58, 59], which contrasts our finding that dietary cholesterol is associated with better β-cell potentiation factor. As there are no previous studies directed at dietary cholesterol and BCF, future studies are necessary to confirm our results.

Higher dietary fiber intake was associated with higher β-cell sensitivity to glucose. Evidence regarding the association between dietary fiber intake and BCF is inconsistent [19, 20, 23], and, so far only focused on early-phase insulin secretion. In line with our findings, one of these studies did not show an association between dietary fiber intake and early-phase insulin secretion [20]. Two other studies [19, 23] revealed a positive association of dietary fiber intake with early-phase insulin secretion, of which one in women only [19]. Such a protective effect of dietary fiber on BCF might be due to delayed glucose uptake from the intestine after dietary fiber intake, resulting in lower postprandial insulin and glucose peaks, and thereby decreased levels of glucotoxicity and a lower burden on β-cells [19, 48, 60]. Furthermore, it was suggested that the high satiating effect of dietary fiber decreases adiposity-associated inflammation, thereby preserving BCF [19, 48, 60]. Significant positive associations of dietary fiber with the other BCF indices might arise when differentiating dietary fiber subtypes, as cereal fibers have much stronger anti-diabetogenic effects than other types of fiber, such as fruit and vegetable fiber [61]. Therefore, future studies should evaluate the association between subcategories of dietary fiber intake and BCF.

Additional analyses were performed in which the disposition indices (DI), another commonly used method to take insulin resistance into account [62], were used as an outcome. The intake of fiber and MUFA were associated with a better DI β-cell glucose sensitivity βs of 0.23 (0.11, 0.34) and 0.25 (0.11, 0.38), respectively, per 10 g of fiber and MUFA intake. Furthermore, *trans* fat intake was associated with a worse DI β-cell glucose sensitivity (β − 0 .14 (− 0.25, − 0.03) per g). Finally, the intake of saturated fat was associated with worse DI β-cell glucose sensitivity and DI C-peptidogenic index per 10 g of intake (βs − 0.22 (− 0.38, − 0.07) and − 0.18 (− 0.38, − 0.01), respectively)(see Supplementary Tables 1 and 2). There are some methodological considerations using the disposition index. First of all, a hyperbolic function between BCF and insulin sensitivity has been suggested as a requirement to apply the disposition index. In our data, such a hyperbolic function was not present, which is in line with some other studies [63, 64]. In addition, artificial relations between indices of BCF and insulin sensitivity may arise when these indices are obtained from insulin and glucose concentrations during a single OGTT [31, 63].

### Strengths and limitations

Strengths of this study are the prospective study design, and the inclusion of participants covering the total glucose metabolism spectrum from NGM to newly diagnosed T2DM, which improves external validity. Other strengths are the simultaneous use of multiple dynamic BCF-indices to assess different aspects of BCF, including both mathematical model parameters and simple BCF measures (66), and the extensive adjustment of our data analyses for potential confounders. However, a methodological consideration is that the CODAM study population is at relatively high risk of developing T2DM, which may reduce external validity. Furthermore, both the exposure and outcome measures were estimated by indirect methods. Even though the FFQ is prone to measurement error, FFQs are well suited to rank individuals according to their intake [65]. However, there may be selective misreporting of fat, sugar, and possibly alcohol, and energy adjustment cannot correct for such selective misreporting. In an attempt to minimize the risk of selective over- or underreporting of specific macronutrients, including sugar and fat, analyses were corrected for BMI; also, persons with previously diagnosed T2DM were excluded from analyses. The FFQ applied in the present study had a reasonable to good reproducibility (> 70%) and validity (60%) for macronutrient intake [34].

As there is no golden standard available to measure BCF, the OGTT was used in this study. Advantages of the OGTT are the relatively good reflection of the physiological insulin secretory response to a glucose load and the provision of a good compromise between accuracy and applicability in an epidemiological study [28, 66].

As BCF is one of the hallmarks of T2DM, the associations of macronutrient intake with multiple aspects of BCF can provide insights into the effects of macronutrients on early abnormalities in glucose metabolism that eventually result in prediabetes and T2DM. As this is the first prospective study evaluating the associations of macronutrient intakes with multiple BCF components, additional observational studies, followed by intervention studies are needed to verify the observed associations. In addition, as nutrients are not being consumed in isolation, an important next step will be to investigate the associations of specific food groups and dietary patterns with BCF.

## Conclusion

In a population at high risk of developing T2DM, MUFA, dietary cholesterol, and dietary fiber intake were associated with a better BCF, whereas polysaccharide and *trans* fat intake were associated with a worse BCF. Our results support the recommendations of several diabetes organizations regarding MUFA, *trans* fat, and dietary fiber intake. As this is the first prospective study evaluating the associations of macronutrient intakes with multiple BCF components, additional studies are needed to verify the observed associations.

## Electronic supplementary material

Below is the link to the electronic supplementary material.

Supplementary material 1 (DOCX 25 KB)

## Abbreviations

BCF: β-cell function
CODAM: Cohort on Diabetes and Atherosclerosis Maastricht
CP: C-peptide
CVD: Cardiovascular disease
DI: Disposition Index
G: Glucose
I: Insulin
NGM: Normal glucose metabolism
OGTT: Oral glucose tolerance test
T2DM: Type 2 diabetes mellitus
