# Supplementary material for: Prospective associations of dietary carbohydrate, fat, and protein intake with β-cell function in the CODAM study
Source: Eur J Nutr. 2018 Mar 10;58(2):597–608. doi: 10.1007/s00394-018-1644-y (PMC6437317; doi:10.1007/s00394-018-1644-y)
Supplement: Supplementary file 1 — Supplementary material 1 (DOCX 25 KB) [file 394_2018_1644_MOESM1_ESM.docx]

| Supplementary table 1: Associations between macronutrient intake and mathematical model disposition indices | | | | | |
| --- | --- | --- | --- | --- | --- |
|  |  |  | DI β-cell glucose sensitivity | DI β-cell rate sensitivity | DI β-cell potentiation ratio |
|  |  | Unit | n=303  β (95% CI) | n=292  β (95% CI) | n=303  β (95% CI) |
| Total carbohydrate | Model 1 | 50g | -0.07 (-0.17-0.03) | -0.02 (-0.18-0.15) | -0.03 (-0.17-0.12) |
|  | Model 2 |  | -0.03 (-0.20-0.14) | -0.03 (-0.28-0.21) | -0.12 (-0.33-0.08) |
| Polysaccharide | Model 1 | 50g | -0.03 (-0.13-0.07) | -0.15 (-0.37-0.07) | -0.03 (-0.22-0.17) |
|  | Model 2 |  | -0.06 (-0.19-0.07) | -0.14 (-0.40-0.13) | -0.07 (-0.29-0.15) |
| Mono- and disaccharide | Model 1 | 50g | -0.07 (-0.17-0.02) | 0.10 (-0.10-0.30) | -0.01 (-0.19-0.17) |
|  | Model 2 |  | -0.02 (-0.16-0.12) | 0.02 (-0.25-0.29) | -0.11 (-0.33-0.11) |
| Fiber | Model 1 | 10g | 0.10 (0.01-0.20) | -0.11 (-0.35-0.12) | 0.07 (-0.14-0.27) |
|  | Model 2 |  | 0.23 (0.11-0.34) | -0.07 (-0.34-0.20) | -0.07 (-0.15-0.30) |
| Total protein | Model 1 | 10g | 0.03 (-0.07-0.13) | -0.03 (-0.14-0.08) | -0.08 (-0.18-0.02) |
|  | Model 2 |  | -0.01 (-0.14-0.12) | 0.01 (-0.11-0.12) | -0.03 (-0.13-0.07) |
| Animal protein | Model 1 | 10g | 0.00 (-0.10-0.09) | -0.01 (-0.10-0.09) | -0.06 (-0.15-0.02) |
|  | Model 2 |  | -0.03 (-0.15-0.08) | 0.00 (-0.11-0.11) | -0.03 (-0.12-0.06) |
| Vegetable protein | Model 1 | 10 g | 0.06 (-0.04-0.16) | -0.08 (-0.32-0.15) | 0.03 (-0.17-0.24) |
|  | Model 2 |  | 0.16 (-0.04-0.36) | 0.09 (-0.31-0.48) | 0.04 (-0.30-0.37) |
| Total fat | Model 1 | 50g | 0.03 (-0.07-0.13) | -0.17 (-0.63-0.30) | -0.24 (-0.65-0.18) |
|  | Model 2 |  | 0.13 (-0.02-0.28) | -0.30 (-0.81-0.21) | -0.21 (-0.63-0.22) |
| Saturated fat | Model 1 | 10g | -0.04 (-0.14-0.05) | -0.03 (-0.22-0.15) | -0.10 (-0.27-0.06) |
|  | Model 2 |  | -0.22 (-0.38- -0.07) | -0.06 (-0.31-0.19) | -0.17 (-0.37-0.04) |
| Trans fat | Model 1 | 1g | -0.10 (-0.20- -0.01) | 0.03 (-0.38-0.43) | -0.06 (-0.42-0.30) |
|  | Model 2 |  | -0.14 (-0.25- -0.03) | 0.16 (-0.28-0.61) | 0.06 (-0.31-0.43) |
| MUFA | Model 1 | 10g | 0.11 (0.01-0.21) | -0.22 (-0.85-0.42) | -0.09 (-0.66-0.48) |
|  | Model 2 |  | 0.25 (0.11-0.38) | -0.36 (-1.03-0.32) | 0.03 (-0.54-0.59) |
| N-3 PUFA | Model 1 | 1g | 0.09 (-0.01-0.19) | -0.03 (-0.28-0.22) | -0.03 (-0.25-0.20) |
|  | Model 2 |  | 0.04 (-0.06-0.14) | -0.02 (-0.28-0.25) | -0.04 (-0.26-0.18) |
| N-6 PUFA | Model 1 | 1g | 0.07 (-0.03-0.16) | -0.02 (-0.05-0.02) | -0.02 (-0.05-0.01) |
|  | Model 2 |  | -0.01 (-0.13-0.11) | -0.02 (-0.06-0.02) | -0.03 (-0.06-0.00) |
| Cholesterol | Model 1 | 100mg | 0.00 (-0.10-0.10) | -0.05 (-0.22-0.14) | -0.11 (-0.27-0.05) |
|  | Model 2 |  | -0.04 (-0.16-0.08) | 0.04 (-0.19-0.26) | 0.04 (-0.15-0.22) |
| *DI, disposition index*  Model 1: adjusted for baseline BCF, age and sex  Model 2: adjusted for model 1 and mean arterial blood pressure, anti-hypertensive medication, lipid-lowering medication, family history of T2DM, total energy intake, and intake of fiber, polysaccharides and MUFA | | | | | |

| Supplementary table 2: Associations between macronutrient intake and simple BCF disposition indices | | | | | |
| --- | --- | --- | --- | --- | --- |
|  |  |  | C-peptidogenic index  n=292 | | CP_AUC_/G_AUC_  n=302 |
|  |  | Unit | β | 95% CI | β (95% CI) |
| Total carbohydrate | Model 1 | 50g | -0.09 (-0.20-0.02) | -0.20, 0.02 | -0.06 (-0.16-0.04) |
|  | Model 2 |  | -0.13 (-0.33-0.07) | -0.33, 0.07 | -0.04 (-0.18-0.11) |
| Polysaccharide | Model 1 | 50g | -0.07 (-0.18-0.04) | -0.18, 0.04 | -0.05 (-0.18-0.08) |
|  | Model 2 |  | -0.03 (-0.17-0.12) | -0.17, 0.12 | -0.05 (-0.21-0.11) |
| Mono- and disaccharide | Model 1 | 50g | -0.06 (-0.17-0.05) | -0.17, 0.05 | -0.04 (-0.16-0.07) |
|  | Model 2 |  | -0.09 (-0.24-0.06) | -0.24, 0.06 | -0.03 (-0.18-0.13) |
| Fiber | Model 1 | 10g | -0.03 (-0.14-0.08) | -0.14, 0.08 | 0.00 (-0.13-0.14) |
|  | Model 2 |  | 0.03 (-0.11-0.16) | -0.11, 0.16 | 0.07 (-0.09-0.23) |
| Total protein | Model 1 | 10g | -0.03 (-0.13-0.07) | -0.13, 0.07 | 0.02 (-0.05-0.08) |
|  | Model 2 |  | 0.01 (-0.10-0.11) | -0.10, 0.11 | 0.01 (-0.05-0.08) |
| Animal protein | Model 1 | 10g | -0.03 (-0.11-0.06) | -0.11, 0.06 | 0.01 (-0.04-0.07) |
|  | Model 2 |  | 0.00 (-0.10-0.09) | -0.10, 0.09 | 0.01 (-0.06-0.07) |
| Vegetable protein | Model 1 | 10 g | 0.03 (-0.18-0.25) | -0.18, 0.25 | 0.00 (-0.13-0.14) |
|  | Model 2 |  | 0.12 (-0.23-0.47) | -0.23, 0.47 | 0.04 (-0.19-0.27) |
| Total fat | Model 1 | 50g | -0.09 (-0.20-0.02) | -0.20, 0.02 | 0.12 (-0.15-0.39) |
|  | Model 2 |  | -0.06 (-0.23-0.10) | -0.23, 0.10 | 0.09 (-0.21-0.39) |
| Saturated fat | Model 1 | 10g | -0.11 (-0.21-0.00) | -0.21, 0.00 | 0.03 (-0.08-0.14) |
|  | Model 2 |  | -0.18 (-0.36- -0.01) | -0.36, -0.01 | -0.03 (-0.18-0.11) |
| Trans fat | Model 1 | 1g | -0.06 (-0.17-0.04) | -0.17, 0.04 | -0.06 (-0.30-0.17) |
|  | Model 2 |  | -0.01 (-0.13-0.12) | -0.13, 0.12 | -0.08 (-0.34-0.17) |
| MUFA | Model 1 | 10g | -0.05 (-0.16-0.06) | -0.16, 0.06 | 0.23 (-0.14-0.60) |
|  | Model 2 |  | 0.03 (-0.12-0.18) | -0.12, 0.18 | 0.20 (-0.19-0.60) |
| N-3 PUFA | Model 1 | 1g | -0.03 (-0.13-0.08) | -0.13, 0.08 | 0.08 (-0.07-0.22) |
|  | Model 2 |  | 0.01 (-0.10-0.12) | -0.10, 0.12 | 0.05 (-0.10-0.21) |
| N-6 PUFA | Model 1 | 1g | -0.04 (-0.15-0.06) | -0.15, 0.06 | 0.00 (-0.02-0.02) |
|  | Model 2 |  | -0.03 (-0.16-0.10) | -0.16, 0.10 | -0.01 (-0.03-0.01) |
| Cholesterol | Model 1 | 100mg | -0.11 (-0.22- -0.01) | -0.22, -0.01 | 0.01 (-0.10-0.12) |
|  | Model 2 |  | -0.02 (-0.16-0.11) | -0.16, 0.11 | 0.00 (-0.13-0.13) |
| *DI, disposition index*  Model 1: adjusted for baseline BCF, age and sex  Model 2: adjusted for model 1 and mean arterial blood pressure, anti-hypertensive medication, lipid-lowering medication, family history of T2DM, total energy intake, and intake of fiber, polysaccharides and MUFA | | | | | |
